# *Microlicia almedae* (Melastomataceae), a new species from the Chapada Diamantina, Bahia, Brazil

**DOI:** 10.3897/phytokeys.276.193021

**Published:** 2026-06-12

**Authors:** Ricardo Pacifico, Ricardo Kriebel

**Affiliations:** 1 São Paulo State University (UNESP), Department of Biology, School of Agricultural and Veterinary Sciences, Jaboticabal, São Paulo, 14884-900, Brazil São Paulo State University (UNESP), Department of Biology São Paulo Brazil; 2 California Academy of Sciences, Institute for Biodiversity Science and Sustainability, Department of Botany, 55 Music Concourse Drive, Golden Gate Park, San Francisco, California 94118-4503, USA California Academy of Sciences, Institute for Biodiversity Science and Sustainability San Francisco United States of America

**Keywords:** Campo rupestre, Lavoisiereae, Pico da Lapa Grande, Serra do Barbado, taxonomy

## Abstract

A new species, *Microlicia almedae*, is described and illustrated from the species-rich campo rupestre vegetation of Chapada Diamantina, Bahia, Brazil. The taxonomic novelty is morphologically related to *M. giuliettiana* and *M. pulchra*, which are also restricted to Chapada Diamantina. *Microlicia almedae* differs from the latter two species in leaf shape and size, hypanthium indumentum, calyx lobe shape and size, and petal size. In addition, *M. almedae* differs from *M. giuliettiana* by its uniformly magenta petals and from *M. pulchra* by its capsules with deciduous columellae. Variation in leaf shape among *M. almedae*, *M. pulchra*, and *M. giuliettiana* was quantified using elliptic Fourier analysis, which revealed near-complete separation among the three species.

## Introduction

According to recent phylogenetic analyses and a revised circumscription, the genus *Microlicia* D.Don was expanded to include species previously recognized in *Chaetostoma* DC., *Lavoisiera* DC., *Stenodon* Naudin, and *Trembleya* DC. (Versiane et al. 2021). Morphologically, *Microlicia* is now delimited within Lavoisiereae DC. by its woody habit and diplostemonous flowers (Versiane et al. 2021; Pacifico and Almeda 2022a; Pacifico et al. 2022a). In addition, its stamens range from isomorphic to dimorphic with tetrasporangiate or polysporangiate anthers that have rostra usually shorter than 1 mm long (Versiane et al. 2021; Pacifico and Almeda 2022a). Its fruits are capsules with basipetal or acropetal dehiscence, and the genus maintains a base chromosome number of *x* = 12 (Versiane et al. 2021; Pacifico and Almeda 2022a). *Microlicia* is largely restricted to Brazil, where over 90% of its approximately 300 species are endemic, inhabiting predominantly campo rupestre and Cerrado ecosystems in the Cadeia do Espinhaço and the Brazilian Central Plateau (Pacifico et al. 2020a; Pacifico and Almeda 2022a). A limited number of species occur in neighboring countries, including Bolivia, Colombia, Guyana, Peru, and Venezuela (Versiane et al. 2020; Pacifico et al. 2020b).

The Chapada Diamantina in central Bahia is recognized as a distinct biogeographic province (Colli-Silva et al. 2019) and a major center of endemism for *Microlicia* (Martins and Almeda 2017; Pacifico et al. 2020a). Intensified research over recent years has resulted in the description of numerous new narrow endemics (see Gali et al. 2022; Pacifico and Almeda 2020, 2022b, 2022c, 2024, 2025, 2026; Pacifico et al. 2022b, 2022c, 2025; Romero et al. 2025). These discoveries emphasize that the region’s botanical richness is still not fully documented, reinforcing the urgent need to protect Bahian campo rupestre sites (Gali et al. 2022; Pacifico and Almeda 2022b, 2026; Pacifico et al. 2025). In December 2025, field expeditions to underexplored areas of the Chapada Diamantina led to the collection of specimens that could not be assigned to any previously described taxa. One of these taxonomic novelties is described and illustrated here.

## Material and methods

### Taxonomic investigations and mapping

This study is based on herbarium specimens housed at CAS, CEPEC, CESJ, JABU, HUEFS, RB, and UEC (acronyms follow Thiers 2026, continuously updated). Comparative morphological characters from *Microlicia pulchra* Pataro & R.Romero and *Microlicia giuliettiana* Almeda & A.B.Martins were obtained from Pataro et al. (2013, 2017) and from specimens at CAS and JABU. The geographic distribution map was prepared using QGIS (Graser et al. 2025) based on information obtained from the labels of the studied exsiccatae. To confirm the municipalities associated with the coordinates provided on specimen labels, the “Validate Spreadsheet” tool on JABOT (https://jabot.jbrj.gov.br/v3/consulta.php) was used.

### Conservation status

The area of occupancy (AOO) and extent of occurrence (EOO) of the species described here were measured using GeoCAT (Bachman et al. 2011) with 2 km^2^ grid cells. The recovered values were then used to suggest a preliminary conservation status based on criterion B as delimited by IUCN (2024).

### Morphometric studies

High-resolution images of 10 fully expanded leaves below the third node from *M. almedae* described here and two hypothesized close relatives, *M. giuliettiana* A.B.Martins & Almeda and *M. pulchra* Pataro & R.Romero, were used to represent variation in leaf morphology of each of the three taxa. Images were converted into binary format with black leaf silhouettes on a white background (see Appendix 1) and saved as JPG files using GIMP 2.10.32 (http://www.gimp.org). The resulting images were imported into the R statistical environment (R Core Team 2026) using the Momocs package (Bonhomme et al. 2014), which converts each outline into a series of *x*–*y* coordinates. Variation in leaf shape was quantified using elliptic Fourier analysis (EFA), a method that describes outline geometry through Fourier coefficients, which are subsequently summarized by principal component analysis (Kuhl and Giardina 1982; Kincaid and Schneider 1983; Claude 2008; Bonhomme et al. 2014). This method has been widely used to quantify leaf shape in many plant groups, including Melastomataceae (Pacifico et al. 2023a, 2023b). Prior to EFA, the rm_sym function in Momocs was used to remove asymmetric variation between the right and left sides of the leaves.

The resulting principal component scores were plotted in a two-dimensional morphospace to evaluate interspecific differences and overlap in leaf shape. The mean shape for each species was also calculated.

## Results

### Morphometrics

**Figure 1.**
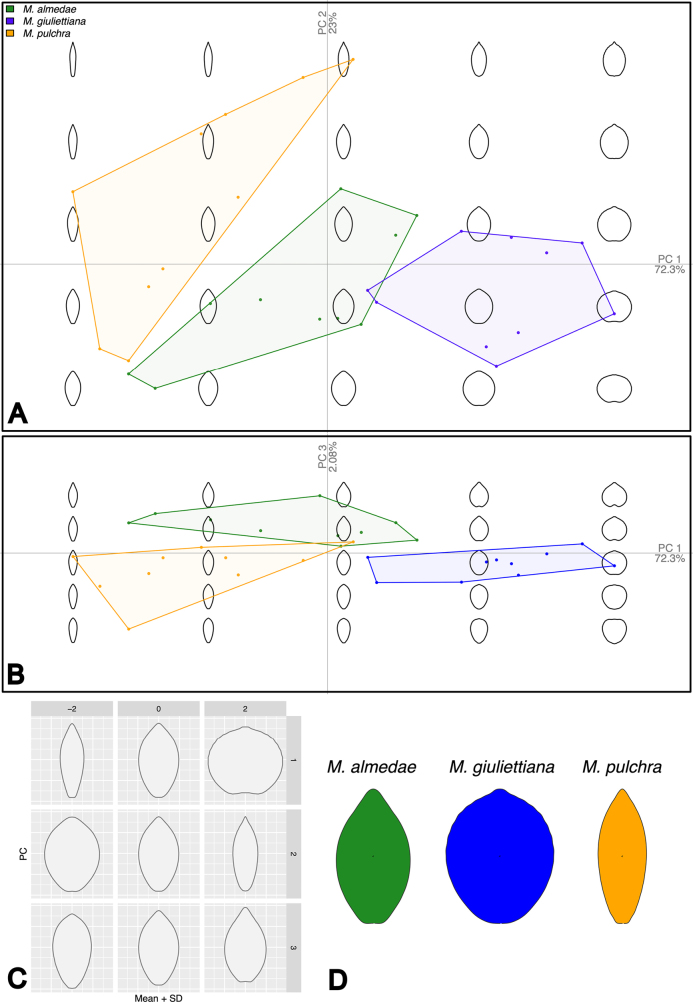
Morphometrics of leaf shape in three species of *Microlicia*. **A**. Morphospace of PC1 and PC2 resulting from ordination of coefficients from elliptic Fourier analysis (EFA) (data points grouped by species); **B**. Morphospace of PC1 and PC3 resulting from EFA (data points grouped by species); **C**. Morphological variation explained in each of the first three principal components. Each row corresponds to variation in each principal component, with the middle shape representing the mean shape and the shapes on each side representing ± 2 standard deviations from the mean shape; **D**. Mean shape of each species in the analysis.

The elliptic Fourier analysis of 30 leaves and subsequent principal component analysis used to summarize the results explained 97.38% of the variation in the first three principal components. The first component accounted for 72.3% of the total variation and explained leaf width, ranging from narrow to broad leaves; the second component accounted for 23% and explained leaf apex shape, ranging from broadly acute to acuminate apices; and the third component accounted for 2% and explained variation in leaf width toward the base or apex, ranging from lanceolate to obovate leaves (Fig. 1A–C).

Grouping the data in morphospace by species revealed nearly complete separation among the three species in leaf shape (Fig. 1A). Narrow-leaved *Microlicia pulchra* was the most distinctive and shared no area in morphospace with the other two taxa. *Microlicia almedae* had leaves that fell between those of *M. pulchra* and *M. giuliettiana*, the latter of which had the broadest leaves of the three taxa. *Microlicia almedae* and *M. giuliettiana* showed a very small area of overlap in leaf shape when looking at PC1 vs. PC2. Plotting the morphospace of PC1 vs. PC3 revealed separation on PC3 between *M. almedae* and *M. giuliettiana*, with the former having broader leaves toward the base and the latter broader leaves toward the apex (Fig. 1B). The mean shape of each species showed that *M. almedae* had elliptic-ovate to ovate leaves, *M. giuliettiana* had broadly ovate to orbicular leaves, and *M. pulchra* had narrowly elliptic leaves (Fig. 1D).

### Taxonomy

#### Microlicia almedae

Taxon classificationPlantaeMyrtalesMelastomataceae

R.B.Pacifico & Kriebel sp. nov.

A6E4B019-37C5-5AFE-B478-8AEF8FD2C600

urn:lsid:ipni.org:names:77381407-1

**Figure 2.**
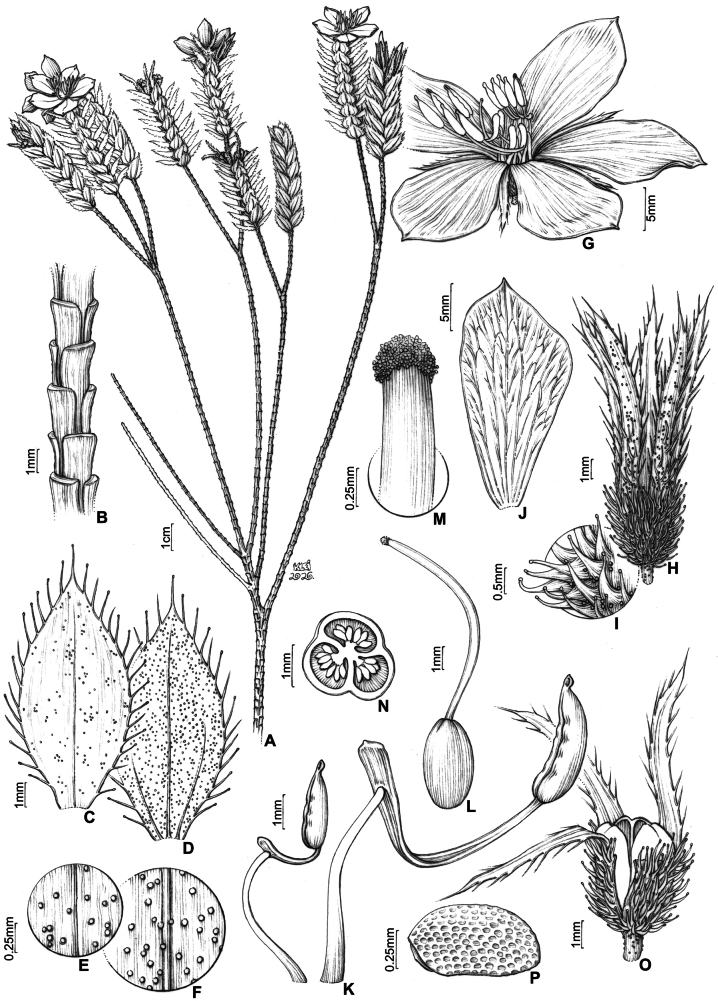
*Microlicia almedae*. **A**. Habit; **B**. Close-up of a branch; **C**. Leaf adaxial surface; **D**. Leaf abaxial surface; **E**. Detail of glandular-punctate adaxial surface; **F**. Detail of glandular-punctate indumentum on abaxial surface; **G**. Flower in lateral view; **H**. Flowering hypanthium and calyx lobes; **I**. Detail of the indumentum on the hypanthium; **J**. Petal in adaxial view; **K**. Antepetalous (left) and antesepalous (right) stamens; **L**. Gynoecium; **M**. Apex of style and stigma; **N**. Ovary in cross-section; **O**. Capsule enveloped by the hypanthium and calyx lobes; **P**. Seed in lateral view. Illustration by Klei Sousa based on *L. Daneu et al. 796* (JABU).

**Figure 3.**
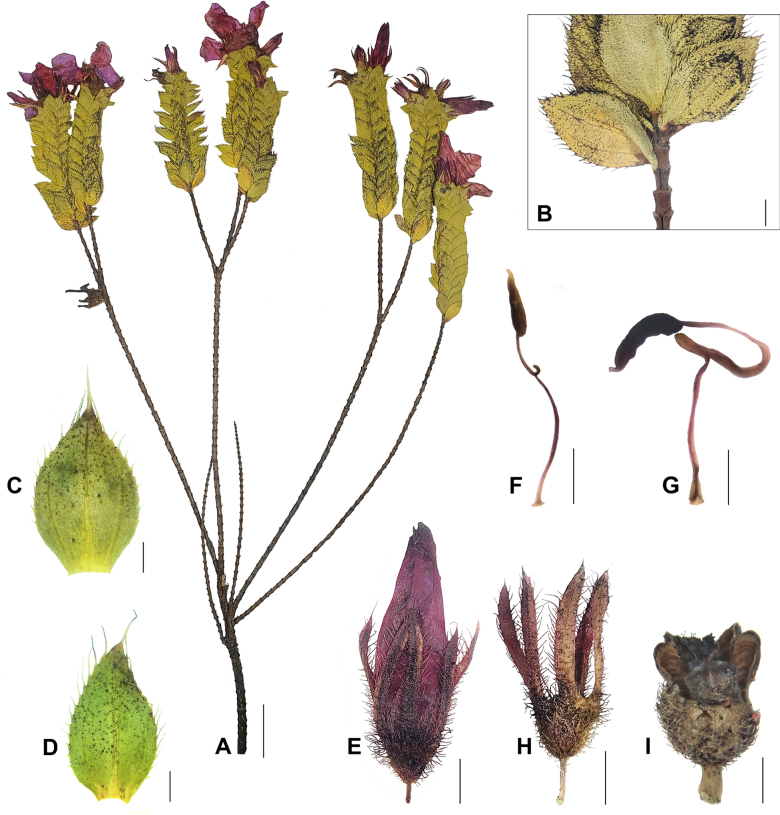
*Microlicia almedae*. **A**. Flowering branch; **B**. Close-up of a branchlet; **C**. Leaf abaxial surface; **D**. Leaf adaxial surface; **E**. Flower bud; **F**. Antepetalous stamen; **G**. Antesepalous stamen; **H**. Flowering hypanthium; **I**. Capsule enveloped by the hypanthium. Photos taken from *L. Daneu et al. 796* (JABU).

**Figure 4.**
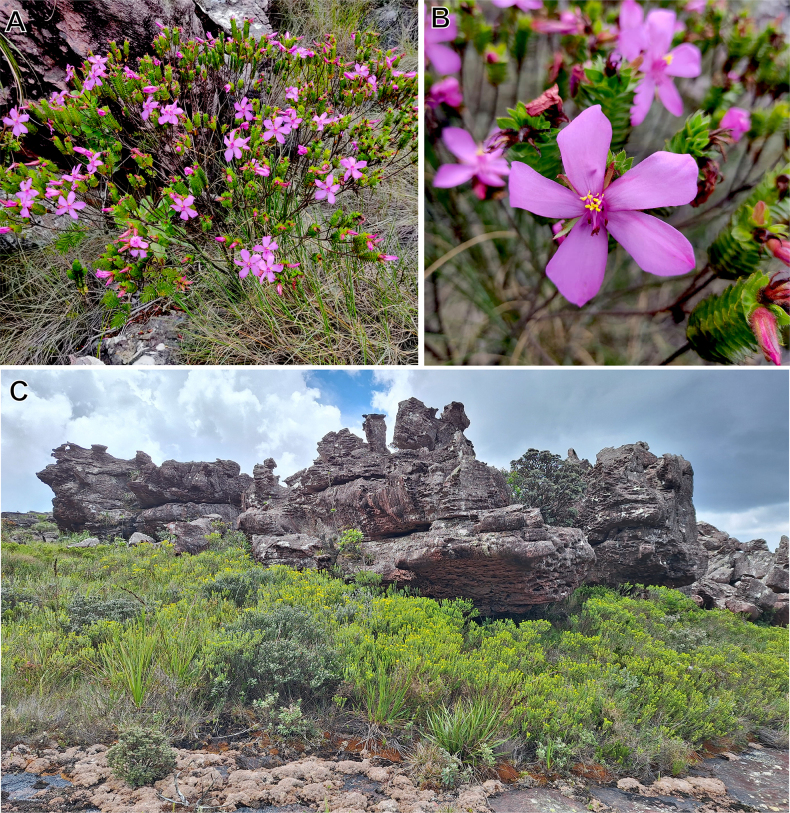
*Microlicia almedae*. **A**. Habit; **B**. Flower; **C**. Landscape with campo rupestre vegetation at Serra do Barbado, the type locality of *M. almedae*. Voucher: *L. Daneu et al. 796* (JABU). Photos: by L. Daneu (**A, B**); by E.A. Ramos (**C**).

**Figure 5.**
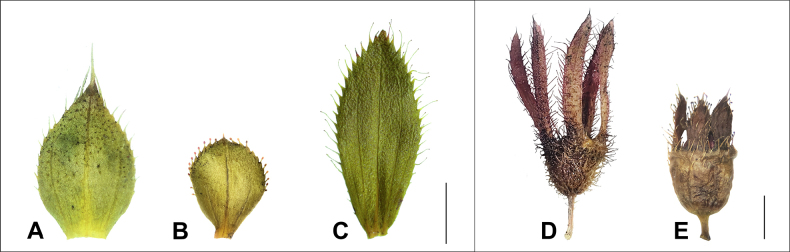
Compared morphological characters among *M. almedae*, *M. giuliettiana*, and *M. pulchra*. **A–C**. Leaf in abaxial view; **A**. *M. almedae*; **B**. *M. giuliettiana*; **C**. *M. pulchra*; **D, E**. Flowering hypanthium; **D**. *M. almedae*; **E**. *M. giuliettiana*. Vouchers: *L. Daneu et al. 796* (JABU) (**A**, **D**); *L. Daneu et al. 861* (JABU) (**B**, **E**); *R. Pacifico et al. 681* (CAS) (**C**).

Figs 2, 3, 4, 5 A, D

##### Type.

Brazil • Bahia: Rio do Pires, Serra do Barbado, acesso por Catolés, ao longo da trilha que liga o Vale dos Frios até a base da Serra do Barbado “pelas costas”, ca. 13°18'18"S, 41°54'04"W, 1650–1825 m, 3 December 2025, fl., fr., *L. Daneu 796, L.C. Gomes & E.A. Ramos* (holotype: JABU barcode JABU00001705!; isotypes: CESJ barcode CESJ085957!, CAS and CEPEC [to be distributed], RB!).

##### Description.

Openly-branched, ***erect shrubs*** 0.6–1.5 m tall. ***Branchlets*** subterete, sparsely glandular-punctate, sulcate on the sides opposite to each other, nodes thickened, stem angles unwinged, internodes 1.5–2.5 mm long. ***Leaves*** sessile, ascendant, decussate, flat, imbricate, semi-amplexicaul; blades 8.9–11.8 × 5.9–7.3 mm, ovate to elliptic ovate, chartaceous, light green and concolored (when fresh), becoming brownish when dry, base slightly attenuate, apex acute to acuminate, both surfaces glandular-punctate, margin inconspicuously serrulate and ciliate with gland-tipped trichomes 0.8–1.2 mm long, the apical trichome to 1.5 mm long, 3-nerved from the base, the venation flat, not prominent on both leaf surfaces, tertiary veins not evident. ***Flowers*** 5-merous, ebracteolate, terminal, clustered at the apex of the branches; ***pedicels*** 2–3 mm long (4–5 mm long in fruit); ***hypanthia*** at anthesis 3.5–5.0 mm long, 4.0–5.5 mm wide at the torus, campanulate, reddish when fresh, becoming brownish when dry, glandular-punctate and densely covered with stout gland-tipped trichomes 0.5–1.5 mm long; ***calyx tubes*** inconspicuous ca. 0.2 mm long; ***calyx lobes*** 7.6–9.5 mm long, 1.3–1.7 mm wide at the base, narrowly lanceolate, reddish as the hypanthia when fresh, becoming brownish when dry, apex acute terminating in a gland-tipped trichome to 1.5 mm long, tardily deciduous, externally glandular-punctate and sparsely covered with gland-tipped trichomes 0.8–1.2 mm long, margin entire and ciliate with similar trichomes; ***petals*** 18–25 × 11–14 mm, oblong-obovate, magenta, margins entire, eciliate, apex shortly acute, both surfaces glabrous; ***stamens*** 10, dimorphic; antesepalous (larger) stamens with filaments 4.2–6.2 mm long, magenta, glabrous, thecae (excluding rostra) 1.9–3.5 × 0.6–1.0 mm, dark purple when fresh, oblong, externally corrugate, polysporangiate, rostra 0.4–0.6 mm long, the ventrally inclined pores 0.2–0.3 mm wide, nearly circular, pedoconnectives 4.7–8.7 mm long, magenta, the appendages 1.4–1.6 mm long, magenta with a yellow apex, apically truncate to emarginate; antepetalous (smaller) stamens with filaments 4.3–5.7 mm long, magenta, glabrous, thecae (excluding rostra) 1.6–2.4 × 0.5–0.7 mm, yellow when fresh, oblong, externally corrugate, polysporangiate, rostra 0.3–0.6 mm long, the ventrally inclined pores ca. 0.2 mm wide, nearly circular, pedoconnectives 1.6–2.2 mm long, the appendages 0.4–0.6 mm long, yellow, apically truncate to emarginate; ***ovaries*** (at anthesis) ca. 4.6 × 3.1 mm, superior, subovoid, glabrous, 3-locular; styles ca. 9.5 mm long, glabrous, sigmoid, stigma truncate. Fruits subovoid loculicidal capsules 5.3–7.4 × 4.7–5.4 mm (at maturity), pale brown, 3-valvate, dehiscent from the apex to the base (basipetal), columellas deciduous, fruiting calyx lobes to 12.5 mm long, lately deciduous. Seeds ca. 0.6 mm long, oblong-reniform, testa foveolate.

##### Recognition.

**Figure 6.**
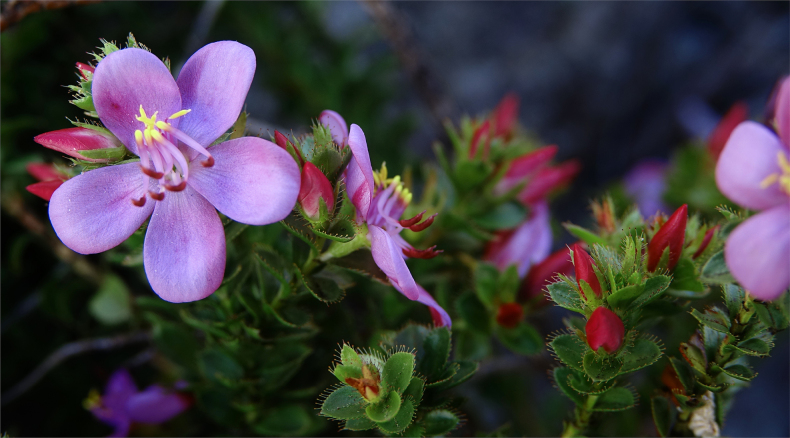
*Microlicia giuliettiana* in Beco do Guiné, Mucugê, Bahia, Brazil. Voucher: F. Almeda et al. 10757 (CAS, CEPEC, HUEM). Photo by F. Almeda.

Differs from *Microlicia giuliettiana* (Figs 5B, 5E, 6) and *M. pulchra* (Fig. 5V) by its ovate to elliptic-ovate leaves (vs. broadly ovate to orbicular in *M. giuliettiana*; narrowly elliptic in *M. pulchra*; Fig. 5), hypanthia covered with gland-tipped trichomes (vs. glabrous in both *M. giuliettiana* and *M. pulchra*), elongated calyx lobes 7.6–9.5 mm long (vs. ca. 4 mm long in *M. giuliettiana*, 3.5–5 mm long in *M. pulchra*), and petals 18–25 mm long (vs. 10–12 mm long in *M. giuliettiana*, 15–16.5 mm long in *M. pulchra*). In addition, *Microlicia almedae* differs from *M. giuliettiana* by its modally larger leaves, 8.9–11.8 × 5.9–7.3 mm (vs. 6–9 × 5–6 mm), and uniformly magenta petals (vs. petals pink with a red band abaxially on the right side; Fig. 6); *M. almedae* also differs from *M. pulchra* in its wider leaves, 5.9–7.3 mm wide (vs. 4–5 mm wide), and capsules with deciduous columellae (vs. persistent).

##### Etymology.

This species is named in honor of the authors’ friend and colleague, Dr. Frank Almeda (1946–), Senior Curator Emeritus of the herbarium of the California Academy of Sciences (CAS), Department of Botany, San Francisco, California, USA. Dr. Almeda is an internationally recognized expert on the family Melastomataceae and has made numerous contributions to plant systematics, including studies on the Brazilian flora and on the tribe Lavoisiereae. Throughout his career, he has also been a generous collaborator and mentor, supporting and inspiring students and researchers from several parts of the world.

##### Distribution, habitat, and phenology.

**Figure 7.**
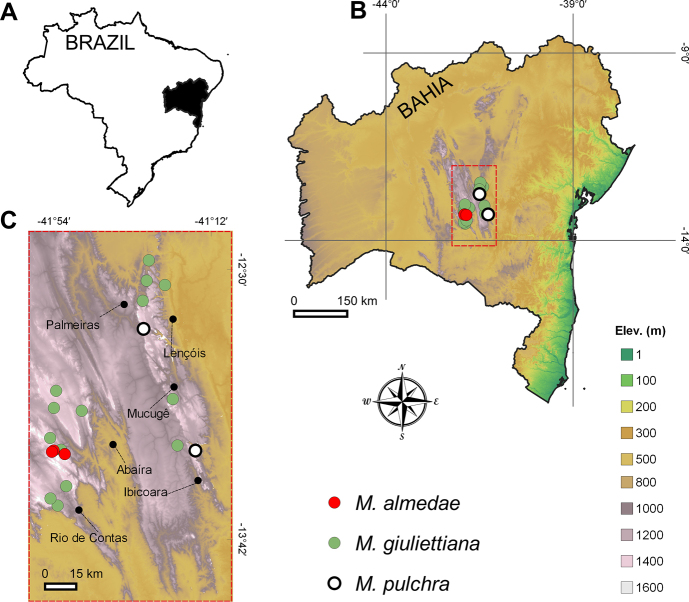
Distributions of *M. almedae* and compared species. **A**. Brazil with the state of Bahia highlighted. **B**. Bahia. **C**. Chapada Diamantina region and the distribution records of *M. almedae*, *M. giuliettiana*, and *M. pulchra*.

*Microlicia almedae* is known only from Serra do Barbado, Pico da Lapa Grande, and Campo do Bicota in the municipalities of Abaíra and Rio do Pires, Chapada Diamantina, Bahia, Brazil (Fig. 7). It grows in campo rupestre exposed to full sun (Fig. 4C) at elevations of ca. 1430–1825 m and was collected flowering in November–December and fruiting in December.

##### Conservation status.

*Microlicia almedae* has an EOO of 2,511 km^2^ and an AOO of 12 km^2^. These values support an Endangered (EN) conservation status under criterion B of the IUCN (2024). All known populations of *M. almedae* occur within a protected area, the Área de Proteção Ambiental da Serra do Barbado. Although this environmental protection area allows certain types of land use and economic activities, it still provides an important level of protection for the populations of *M. almedae* and many other narrowly endemic plants (e.g., Hind 1995; Woodgyer and Zappi 2005; Salariato et al. 2011; Saavedra et al. 2014; Giulietti and Silva 2016; Pacifico and Almeda 2018, 2022b, 2025; Carmo et al. 2022).

##### Additional specimens examined (paratypes).

Brazil • Bahia: Abaíra. Catolés, Campo do Bicota, próximo a subida que dá acesso ao campo, [ca. 13°19'17.32"S, 41°51'6.05"W], [ca. 1430 m], 28 November 1999, fl., *A.S. Conceição 462 & G.L. Campos* (HUEFS, UEC!). • Rio do Pires. Pico da Lapa Grande, acesso por Catolés passando pelo Vale dos Frios, coletada ao longo da trilha da base ao topo do pico, ca. 13°18'39"S, 41°54'34"W, 1650–1825 m, 4 December 2025, fl., fr., L. *Daneu 801, L.C. Gomes & E.A. Ramos* (CAS!, JABU!, RB!).

## Supplementary Material

XML Treatment for Microlicia almedae

## Figures and Tables

**Figure A1.**
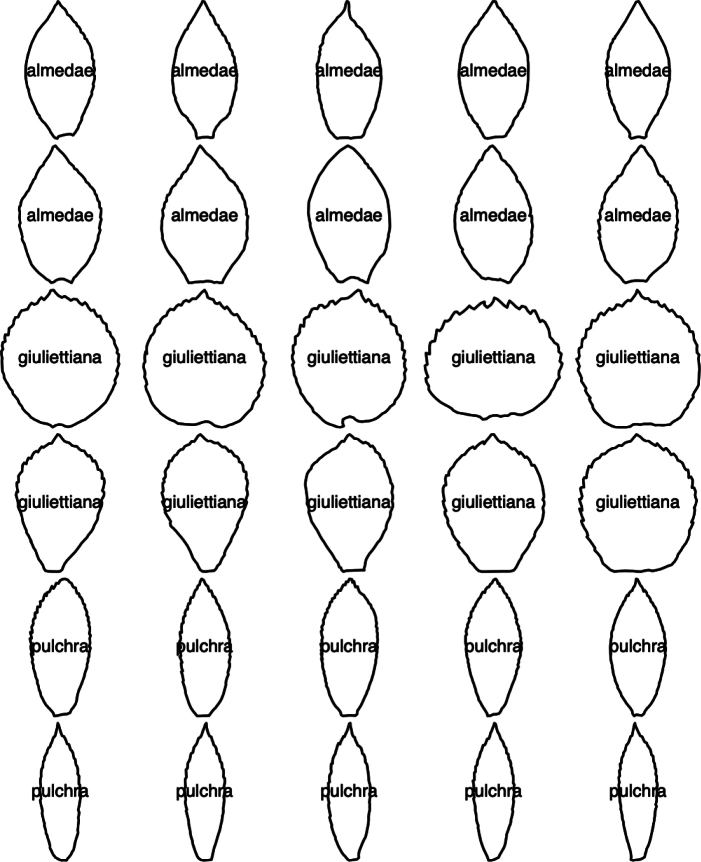
Outlines of leaves of *Microlicia almedae*, *M. giuliettiana*, and *M. pulchra*, which were used in the elliptic Fourier analysis of leaf shape.

## Additional information

### Artificial Intelligence (AI) use

The authors accept full responsibility for the content of the manuscript, including the disclosure of any use of AI.

No AI tools were used in the preparation of this manuscript.

### Author ORCIDs

R. Pacifico https://orcid.org/0000-0001-9566-5344

R. Kriebel https://orcid.org/0000-0002-1138-7533

### Data availability

All of the data that support the findings of this study are available in the main text.
